# Identification and functional characterization of type II toxin/antitoxin systems in *Aggregatibacter actinomycetemcomitans*

**DOI:** 10.1111/omi.12215

**Published:** 2018-02-20

**Authors:** B. Schneider, W. Weigel, M. Sztukowska, D.R. Demuth

**Affiliations:** ^1^ Department of Oral Immunology and Infectious Disease University of Louisville School of Dentistry Louisville KY USA; ^2^ Present address: Hemholz Centre for Infection Research Campus Braunschweig Imhoffenstrasse 7 38124 Braunschweig Germany

**Keywords:** *Aggregatibacter actinomycetemcomitans*, biofilm, RelBE, toxin/antitoxin

## Abstract

Type II toxin/antitoxin (TA) systems contribute to the formation of persister cells and biofilm formation for many organisms. *Aggregatibacter actinomycetemcomitans* thrives in the complex oral microbial community subjected to continual environmental flux. Little is known regarding the presence and function of type II TA systems in this organism or their contribution to adaptation and persistence in the biofilm. We identified 11 TA systems that are conserved across all seven serotypes of *A. actinomycetemcomitans* and represent the RelBE, MazEF and HipAB families of type II TA systems. The systems selectively responded to various environmental conditions that exist in the oral cavity. Two putative RelBE‐like TA systems, D11S_1194‐1195 and D11S_1718‐1719 were induced in response to low pH and deletion of D11S_1718‐1719 significantly reduced metabolic activity of stationary phase *A. actinomycetemcomitans* cells upon prolonged exposure to acidic conditions. The deletion mutant also exhibited reduced biofilm biomass when cultured under acidic conditions. The D11S_1194 and D11S_1718 toxin proteins inhibited in vitro translation of dihydrofolate reductase (DHFR) and degraded ribosome‐associated, but not free, MS2 virus RNA. In contrast, the corresponding antitoxins (D11S_1195 and D11S_1719), or equimolar mixtures of toxin and antitoxin, had no effect on DHFR production or RNA degradation. Together, these results suggest that D11S_1194‐1195 and D11S_1718‐1719 are RelBE‐like type II TA systems that are activated under acidic conditions and may function to cleave ribosome‐associated mRNA to inhibit translation in *A. actinomycetemcomitans*. In vivo, these systems may facilitate *A. actinomycetemcomitans* adaptation and persistence in acidic local environments in the dental biofilm.

## 1 INTRODUCTION

*Aggregatibacter actinomycetemcomitans* is a non‐motile, facultative Gram‐negative bacterium in the *Pasteurellaceae* family that is most commonly found in the oral cavity. It is implicated in the etiology of aggressive and chronic periodontitis, but is also associated with extra‐oral infections such as infective endocarditis, soft‐tissue abscesses, meningitis, pneumonia, septicemia, urinary tract infections and osteomyelitis.^1^, ^2^, ^3^, ^4^, ^5^, ^6^ *Aggregatibacter actinomycetemcomitans* expresses a variety of virulence factors that facilitate its persistence and survival in the oral cavity and the activity of these factors contributes to increased inflammation, immune suppression, tissue destruction and alveolar bone resorption.^7^, ^8^ The molecular mechanisms by which *A. actinomycetemcomitans* successfully colonizes and persists in the dental biofilm, and its ability to disseminate from this niche to other organs of the body, have not been well defined.

The dental biofilm is a complex and dynamic microbial community that is comprised of up to 700 different species of bacteria.^9^, ^10^, ^11^, ^12^, ^13^, ^14^ This biofilm is the prime etiological agent of three common oral diseases in humans: dental caries, gingivitis and periodontal disease.^1^, ^15^, ^16^ The progression of these diseases is associated with major shifts in microbial populations in the oral biofilm and diseased sites often exhibit increased populations of pathogenic species relative to healthy sites in the oral cavity.^15^, ^16^, ^17^ The stimuli that contribute to these population shifts have not been well characterized but the oral cavity is subject to continual environmental flux, including changes in pH, temperature, osmolarity and nutrient supply. Oral bacteria rapidly detect and respond to these environmental fluctuations, allowing them to successfully coexist and thrive in the oral cavity.^16^, ^18^, ^19^ A variety of mechanisms contribute to adaptation to environmental flux but there is growing evidence that the activity of toxin/antitoxin (TA) systems play an important role in adapting to and persisting under conditions of environmental stress.^20^

The TA systems comprise a variety of genetic elements classified in six different families or types based on the mechanism of action of the antitoxins and are encoded on both plasmids and the bacterial chomosome.^21^, ^22^, ^23^, ^24^ Type II TA systems have been most extensively studied and encode protein toxins and antitoxins. Interaction of the antitoxin with the toxin inhibits toxin activity^25^ and in many cases, the antitoxin and/or the toxin‐antitoxin complex can also function as a transcriptional repressor to autoregulate their expression.^26^, ^27^ Under conditions of environmental stress, cellular proteases (eg, Lon and ClpXP) are activated, which degrade the labile antitoxin, activating the toxin. Type II toxins target important physiological functions such as translation, DNA replication, cell wall synthesis and the assembly of cytoskeletal proteins during cell division, leading to growth arrest.^21^ Many type II toxins function as ribonucleases that cleave their target mRNAs in either a ribosome‐dependent or independent manner.^22^ Very little is known about the presence and function of *A. actinomycetemcomitans* type II TA systems and how they may contribute to adaptation and persistence of the organism in the dental biofilm. In this study, we identified 11 operons in the *A. actinomycetemcomitans* D11S genome that encode putative functional type II TA systems and show that these systems respond selectively to various environmental conditions. Two RelBE‐like systems were activated under acidic growth conditions and deletion of these systems resulted in reduced metabolic activity of *A. actinomycetemcomitans* in stationary phase. Finally, we demonstrate that both of these TA systems inhibit translation and function as ribosome‐dependent ribonucleases.

## 2 MATERIALS AND METHODS

### 2.1 Bacterial strains and plasmids

The bacterial strains and plasmids used in this study are listed in the Supplementary material (Tables S1 and S2), respectively. Luria‐Bertani (LB) broth or LB agar (LB broth plus 1.5% agar) was used for the propagation or plating of *Escherichia coli*. Bacteria were grown at 37°C with shaking for broth cultures or under microaerophilic conditions in an atmosphere of 5% CO_2_ for plates. *Aggregatibacter actinomycetemcomitans* strain 652 was routinely cultured in brain‐heart infusion (BHI) broth or BHI agar (BHI plus 1.5% agar) under microaerophilic conditions, either in a candle jar for plates or as standing broth cultures in an atmosphere of 5% CO_2_. For the construction of gene deletion mutants, SOC medium (2% tryptone, 0.5% yeast extract, 10 mm NaCl, 2.5 mm KCl, 10 mm MgCl_2_ and 20 mm glucose), TYE (1% tryptone and 0.5% yeast extract) or TYE agar (TYE plus 2% agar) were routinely used for the propagation or plating of *A. actinomycetemcomitans*. For some experiments, *A. actinomycetemcomitans* was grown in a chemically defined medium as described by Socransky et al.^28^ with some modifications (see Supplementary material, Table S3). When necessary for plasmid selection, medium was supplemented either with 25 μg mL^−1^ kanamycin, 50 μg mL^−1^ spectinomycin, 12.5 μg mL^−1^ tetracycline, 50 μg mL^−1^ ampicillin, 40% sucrose or 1 mm isopropyl B‐d‐1‐thiogalactopyranoside (IPTG).

### 2.2 Identification of putative TA systems

Putative type II TA systems in *A. actinomycetemcomitans* were identified by two methods. First, the genome of *A. actinomycetemcomitans* strain D11S‐1, serotype c^29^ was probed for sequence similarity to known *E. coli* TA systems using the protein basic local alignment search tool (pBLAST, https://www.ncbi.nlm.nih.gov). The sequences for known *E. coli* toxins that were used in these searches are listed in the Supplementary material (Table S4). In addition, the entire genome of *A. actinomycetemcomitans* D11S‐1 was examined using TAfinder (http://202.120.12.133/TAfinder/TAfinder.php) to identify operons that were not previously identified in the BLAST searches described above. TAfinder detects type II TA loci mainly based on sequence alignments and conserved domain searches against a database of TA families. The *A. actinomycetemcomitans* peptide sequences identified as putative TA systems in strain D11S‐1 were subsequently used to probe 33 other *A. actinomycetemcomitans* genome sequences that were present in the NCBI database (see Supplemental material, Table S5) that represent all seven *A. actinomycetemcomitans* serotypes.

### 2.3 Expression of putative TA systems under environmental stress

The expression of the putative TA systems was determined under various environmental stress conditions using real‐time polymerase chain reaction (PCR). Cells were grown to mid‐exponential phase in chemically defined medium supplemented with 20 mm glucose or 20 mm lactate^30^ at 37°C and were then exposed to various environmental conditions for 20 min. Environmental conditions examined included: acidic pH (pH 5.0), oxidative stress (0.1% hydrogen peroxide), microaerophilic conditions (5% CO_2_), elevated temperature (39°C), anaerobic conditions (10% H_2_, 10% CO_2_, 80% N_2_), iron limitation (250 μm bipyridyl), and reduced temperature (30°C). Bacteria were harvested and RNA was isolated using the cesium chloride step‐gradient method as described by Reddy and Gilman.^31^ RNA was reverse transcribed to cDNA using random primers provided in the cDNA synthesis kit (Quanta Bio, Beverley, MA), and the resulting cDNA was used in SYBR Green real‐time PCR using primers for the TA system as recommended by the manufacturer (Quanta Bio). Data were analyzed using the ΔΔCt method and fold change expression was determined by normalizing the results to the levels of an unstressed control (5S rRNA was used for normalization). The putative TA systems that are co‐expressed with a third gene (D11S_0469‐470 and D11S_2094‐2095) were excluded in these analyses.

### 2.4 Construction of isogenic deletion mutants

The generation of markerless deletion mutations was carried out as described by Juárez‐Rodríguez et al^32^ with some modifications. PCR fragments of the upstream and downstream flanking regions of the gene targeted for deletion were amplified by PCR and ligated into the pJT1 suicide vector.^33^ Primers used in these reactions are shown in the Supplementary material (Table S6). After transformation of *E. coli* with the resulting ligation mixture, recombinant colonies exhibiting spectinomycin resistance were analyzed by restriction digestion and gel electrophoresis to confirm the presence of the desired insert fragments. Inserts were then further confirmed by DNA sequencing. The resulting plasmids were introduced into *A. actinomycetemcomitans* by electroporation. Electroporated cells were recovered in SOC broth for 5 h before being plated on BHI agar plates supplemented with spectinomycin. Resistant colonies were selected, cultured again in BHI broth supplemented with spectinomycin and then passaged overnight twice in BHI broth lacking antibiotic. Samples were then inoculated into TYE broth supplemented with IPTG to induce the expression of levansucrase encoded by *sacB* carried on the pJT1 plasmid vector. Levansucrase forms long levan chains from sucrose and is lethal to Gram‐negative bacteria in the presence of sucrose. Cultures were then serially diluted and plated on TYE agar supplemented with IPTG and sucrose. A minimum of 100 colonies were replica plated onto TYE agar supplemented with IPTG and sucrose and BHI agar supplemented with spectinomycin. Colonies that were sucrose resistant and antibiotic sensitive were selected for PCR confirmation that the genes of interest were deleted. Colonies that were positive for gene deletion were further confirmed by DNA sequencing. No differences in planktonic growth of the mutant and wild‐type strains were observed.

### 2.5 Measurement of metabolic activity

To determine the metabolic activity of isogenic mutants, 3‐(4,5‐dimethylthiazol‐2‐yl)‐2,5‐diphenyl tetrazolium bromide (MTT) was used as described by Wang et al.^34^ with some modifications. Cultures were grown at 37°C to stationary phase, removed from the incubator (*t* = 0) and incubated at room temperature for 5 days. At various time points, 3 mL of the culture was removed, centrifuged at 5000 *g* and suspended in fresh BHI broth containing 2.07 mm MTT. Cultures were then incubated for 2 h (approximately one doubling period) before being harvested. Metabolically active cells reduce MTT via NADH^+^/NADPH^+^‐dependent oxidoreductase enzymes in the electron transport chain to form water‐insoluble formazan.^35^ The resulting formazan crystals were dissolved in dimethyl sulfoxide and absorbance was measured at 550 nm.

### 2.6 Growth and analysis of static biofilms

Static biofilms were grown in multi‐well tissue culture plates.^36^, ^37^, ^38^ The *A. actinomycetemcomitans* cultures were grown overnight in BHI broth and the optical density at 600 nm (OD_600_) was measured. To form mature *A. actinomycetemcomitans* biofilms, cells were diluted into fresh BHI broth to a final OD_600_ of 0.005 and this dilution was used to inoculate the plate wells. Cultures were incubated at 37°C for 72 h and the resulting biofilms were then supplied with fresh BHI broth that was adjusted from pH 5.0—pH 8.0 to represent the range of conditions that might typically exist in the gingival pocket.^39^, ^40^ The biofilms were subsequently incubated for an additional 24 h, rinsed gently with sterile water and stained with 0.1% crystal violet. After staining, cells were rinsed with sterile water until the water was clear, crystal violet was solubilized with 30% acetic acid and the absorbance at OD_570_ was measured.

### 2.7 Statistical analysis

All assays were carried out in triplicate and data were analyzed using the unpaired *t*‐test with statistical significance defined as *P* ≤ .05.

### 2.8 Functional analysis of putative TA systems

Peptides representing the toxin and antitoxin proteins of each TA system (sequences are shown in the Supplementary material, Table S7) were chemically synthesized (Biosynthesis, Inc., Lewisville, TX) at > 90% purity. Synthetic peptides were dissolved in 0.05 m phosphate buffer, pH 7.8, containing 300 mm NaCl and 0.01% trifluoroacetic acid and peptide purity and size was confirmed using a NuPAGE Bis‐Tris SDS‐PAGE gel (Thermo Fisher Scientific, Waltham, MA). Samples were prepared in NuPAGE SDS sample buffer according to the recommendation of the manufacturer and gels were electrophoresed using NuPAGE MES Buffer in an XCell SureLock MiniCell. To test the ability of the synthetic toxins and antitoxins to interfere with protein translation, a cell‐free protein synthesis system was used. Toxin (200 ng), antitoxin (200 ng) or an equimolar mixture of both proteins were added to the PURExpress In Vitro Protein Synthesis Kit (New England BioLabs, Ipswich, MA) and translation of dihydrofolate reductase (DHFR) from the control plasmid supplied in the kit was examined. Reactions were incubated in a thermocycler at 37°C for 2 h and 5 μL of the reaction was used for analysis on a Tris‐Glycine SDS‐PAGE gel according to the manufacturer's instructions. All protein gels were stained with AcquaStain 1‐Step Protein Gel Stain (Bulldog Bio, Portsmouth, NH) overnight and destained with sterile water for 20 min before imaging. To determine if the toxin and antitoxin proteins function to degrade mRNA in the presence of ribosomes, MS2 bacteriophage genomic RNA (Roche, Basel, Switzerland) was mixed with toxin (200 ng), antitoxin (200 ng) or an equimolar mixture of both in the presence and absence of ribosomes. Reactions were incubated at 37°C for 30 min and the reaction was stopped with RNA loading dye containing 47.5% formamide (New England BioLabs). Samples were denatured at 85°C for 15 min before analysis on a TBE‐Urea gel according to the manufacturer's recommendation. Gels were rinsed in diethyl pyrocarbonate‐treated water for 10 min and were stained for 20 min with ethidium bromide and rinsed twice with diethyl pyrocarbonate‐treated water before imaging.

## 3 RESULTS

### 3.1 Identification of type II TA systems in *A. actinomycetemcomitans*

Type II TA systems encode two small proteins (each between ~40 and 200 amino acids) that can regulate bacterial cell function including the formation of biofilms and the cellular response to various environmental stresses (eg, antibiotic treatment, starvation). To determine if the *A. actinomycetemcomitans* genome encodes type II TA systems, peptide sequences of known TA systems that are present in *E. coli* were used to perform BLAST searches of the *A. actinomycetemcomitans* D11S‐1 genome. As shown in Table 1, this search identified nine putative type II TA systems. Two of these TA operons exhibit sequence similarity to the MazEF‐family of TA systems, which function as site‐specific endoribonucleases that cleave mRNA independent of ribosome binding, resulting in inhibition of translation. One *A. actinomycetemcomitans* TA system was related to the HipAB‐family, which acts as a serine/threonine kinase targeting tRNA to inhibit translation. The remaining six putative type II TA systems of *A. actinomycetemcomitans* were related to the RelBE‐family, which function as ribosome‐dependent endoribonucleases to cleave mRNA.

**Table 1:** Type II toxin/antitoxin systems in *Aggregatibacter actinomycetemcomitans*

Toxin	Antitoxin	Gene identity	Family	Total score	Query cover	E value	Maximum identity
mazF	mazE	D11S_0905‐0906	mazEF	101	92%	2e‐28	33%
chpBK	chpBI	D11S_0919‐0920	mazEF	88.6	93%	2e‐23	42%
hipA	hipB	D11S_1069‐1070	hipAB	108	21%	1e‐28	45%
yafQ	dinJ	D11S_1194‐1195	relBE	89.7	95%	2e‐24	36%
yoeB	yafM	D11S‐1718‐1719	relBE	125	100%	1e‐38	63%
relE	relB	D11S_1798‐1799	relBE	112	94%	6e‐33	57%
relE	relB	D11S_2133‐2134	relBE^a^	44.7	88%	2e‐7	12%
relE	relB	D11S_1417‐1418	relBE	102	98%	3e‐30	54%
relE	relB	D11S_1144‐1145	relBE	149	97%	2e‐46	59%
—	—	D11S_1023‐1024	—	—	—	—	—
—	—	D11S_0150‐0151	a	—	—	—	—
—	—	D11S_0499‐0500	a	—	—	—	—
—	—	D11S_0469‐0470	b	—	—	—	—
—	—	D11S_2094‐2095	b	—	—	—	—

a Sequence analysis indicated that this operon contained a toxin and/or antitoxin pseudogene.

b Identified by TAfinder but present in a three‐gene operon with a non‐toxin/antitoxin (TA) ‐related gene (see text).

As approximately 32% of the genes in the *A. actinomycetemcomitans* genome have not been assigned a function, it is possible that *A. actinomycetemcomitans* encodes additional type II TA systems that may not be related to known *E. coli* TA system sequences. Hence, the entire *A. actinomycetemcomitans* genome was further examined using TAfinder, which detects type II TA loci using both sequence alignment and conserved domain searches against a database of diverse TA families. As shown in Table 1, TAfinder identified five additional putative type II TA systems. None of these exhibited significant sequence similarity to *E. coli* TA systems. In addition, D11S_0469‐470 and D11S_2094‐2095 were unique in that each operon contained a third open reading frame encoding a gene unrelated to toxins or antitoxins, diadenosine tetraphosphatase and *O*‐succinylbenzoate synthase, respectively. Finally, sequence analysis suggested that three of the putative TA systems may contain pseudogenes (indicated by an asterisk in Table 1). Hence, the *A. actinomycetemcomitans* D11S‐1 genome encodes at least 14 putative type II TA loci, three of which may contain pseudogenes and may be non‐functional.

To determine the extent to which the type II TA systems identified in serotype c strain D11S‐1 are conserved across other *A. actinomycetemcomitans* serotypes, peptide sequences representing each of the TA systems identified in D11S‐1 were compared with 33 other *A. actinomycetemcomitans* genomes contained in the sequence database. As shown in the Supplementary material (Tables S8a–S8e), most of the D11S‐1 TA systems are highly conserved across all of the other *A. actinomycetemcomitans* serotypes. Two exceptions were D11S_150‐151 and D11S_1069‐1070. A complete D11S_1069‐1070 operon was present in only three of the 33 genomes examined (D11S‐1, SC383s and ANH9381); the other strains either lacked the toxin open reading frame or possessed a truncated gene, but interestingly most contained the antitoxin gene. D11S_150‐151 was present in all serotype a, b, d, f and g strains but was absent in the serotype c organisms except for D11S‐1, which possessed a putative pseudogene. For many of the strains across all of the serotypes, some TA systems were present in multiple copies. For example, serotype b strain RhAA1 possesses at least 19 type II TA systems including two copies of D11S_0469‐0470, D11S_0905‐0906, D11S_1418‐1419 and D11S_2133‐2134, and three copies of D11S_1023‐1024 and D11S_1798‐1799, however this strain also lacked D11S_1069‐1070, D11S_1194‐1195 and D11S_2094‐2095. Finally, PCR using *A. actinomycetemcomitans* 652 (serotype c) DNA as a template indicated that all of the D11S‐1 TA systems were present in this strain (not shown). Although a complete genome sequence is not yet available, strain 652 has been extensively characterized in our laboratory and was used for the functional characterization of the *A. actinomycetemcomitans* TA systems described below. Overall, these results suggest that the TA systems identified in the D11S‐1 genome are highly conserved in all *A. actinomycetemcomitans* serotypes but that D11S_150‐151 and D11S_1069‐1070 were more limited in distribution than the other type II TA systems.

### 3.2 Putative TA systems in *A. actinomycetemcomitans* respond to environmental stress

**Figure 1:**
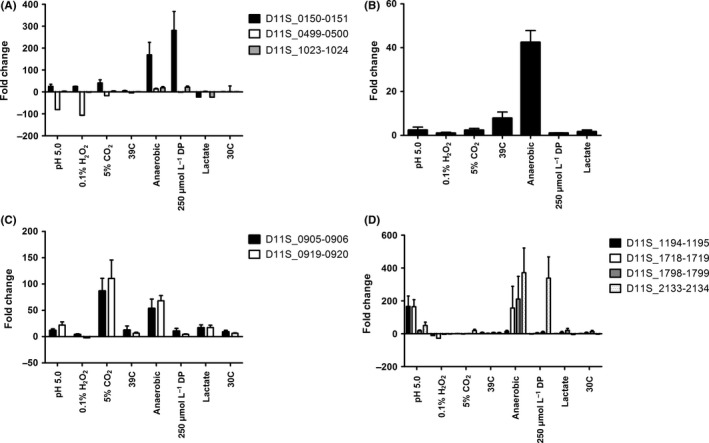
Induction of type II toxin‐antitoxin (TA) system expression in *Aggregatibacter actinomycetemcomitans* exposed to various environmental conditions. A, TA systems D11S_0150‐0151, D11S_0499‐500 and D11S_1023‐1024; B, D11S_1069‐1070 related to the HipAB family of TA systems; C, D11S_0905‐0906 and D11S_0919‐0920 related to the MazEF family of TA systems; and D, D11S_1194‐1195, D11S_1718‐1719, D11S_1798‐1799 and D11S_2133‐2134 related to the RelBE family of TA systems

For many of the known type II TA systems, the complex of the toxin and antitoxin proteins functions as a repressor that autoregulates its own operon expression. Under environmental stress, proteases such as Lon are activated and degrade the labile antitoxin, activating the toxin and de‐repressing operon expression. To assess the effect of environmental stress on *A. actinomycetemcomitans* TA expression, cells were exposed to various stress conditions and TA expression was determined by real‐time PCR. As shown in Figure 1A, the expression of D11S_0150‐0151 and D11S_1023‐1024 was induced to the greatest extent when cells were cultured under anaerobic conditions or under iron‐limited conditions (250 μm dipyridyl), and to a lesser extent when cells were cultured at pH 5.0 or under microaerophilic conditions. Interestingly, D11S_499‐500 was down‐regulated at pH 5.0 or when cells were cultured in the presence of H_2_O_2_. As shown in Figure 1B, the HipAB‐like D11S_1069‐1070 TA system exhibited the greatest increase in expression when cells were cultured anaerobically.

The two MazEF‐related TA systems exhibit a similar pattern of expression and both are significantly induced under microaerophilic and anaerobic growth conditions and to a lesser extent when cells were cultured at pH 5.0 or when lactate is the carbon course (Figure 1C). The RelBE‐related TA systems responded mainly to growth under acidic or anaerobic conditions. As shown in Figure 1D, D11S_1194‐1195 and D11S_1718‐1719 were strongly induced when cells were cultured at pH 5.0 and D11S_1718‐1719 and D11s_1798‐1799 were significantly induced under anaerobic conditions.

Together, these results clearly show that the putative TA systems respond differentially to environmental stress and that specific TA systems preferentially responded under certain stress conditions. Seven of the 11 systems comprising members of each of the TA families were induced under anaerobic conditions, suggesting that a high degree of functional redundancy may exist in the TA systems contributing to adaptation to anaerobic growth. In contrast, only two systems were significantly induced when cultures were either grown under microaerophilic conditions, at pH 5.0, or in the presence of dipyridyl, which suggests that some TA systems are functionally more specific. Only one TA system, D11S_2133‐2134, did not respond to any of the environmental stress conditions that were tested.

### 3.3 Functional characterization of D11S_1194‐1195 and D11S_1718‐1719

During growth of *A. actinomycetemcomitans* in broth culture, the pH of the medium drops from an initial pH 7.5 to pH 5.5‐6.0 when the culture is in stationary phase. Bhattacharjee et al^41^ previously showed that if the pH of spent medium was readjusted to neutral, *A. actinomycetemcomitans* continued to grow without requiring any other additives. This suggests that entrance into stationary phase may be dependent on environmental pH rather than depletion of available nutrients in the medium. Two of the putative RelBE‐like TA systems, D11S_1194‐1195 and D11S_1718‐1719, were highly induced when *A. actinomycetemcomitans* was cultured at pH 5.0 (Figure 1D), suggesting that these systems may contribute to overall fitness of the organism at reduced pH. These two TA systems were selected for further study. A gene deletion mutant of each TA system was constructed and metabolic activity of stationary phase cultures of these strains at pH 5.0 were compared with the wild‐type strain.

**Figure 2:**
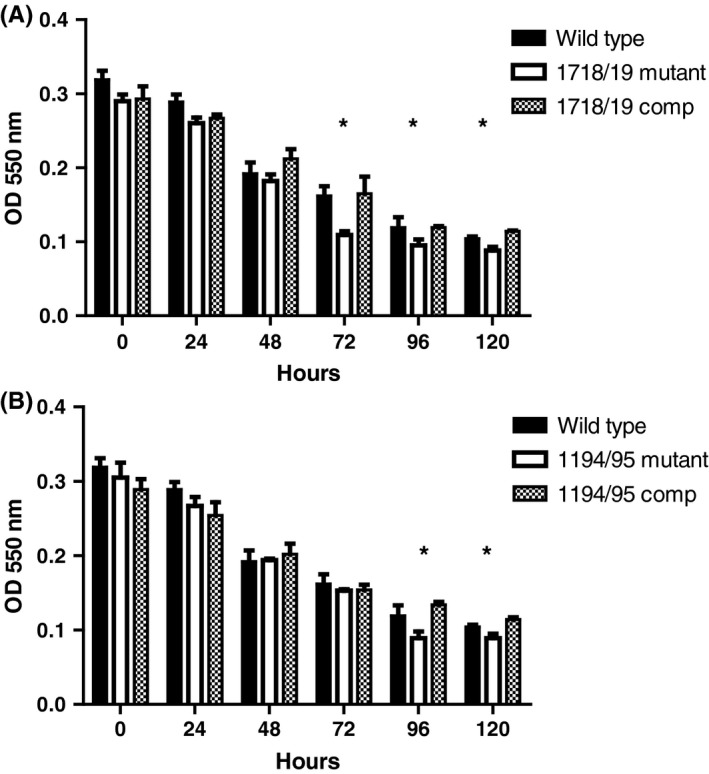
Metabolic activity of stationary phase *Aggregatibacter actinomycetemcomitans* wild‐type, toxin‐antitoxin (TA) system deletion mutants, and complemented strain cultures. Metabolic activity was measured using 3‐(4,5‐dimethylthiazol‐2‐yl)‐2,5‐diphenyl tetrazolium bromide (MTT) as described in the Materials and methods. Asterisks indicate the time points where metabolic activity of the mutant strain was significantly reduced relative to the wild‐type (*P *≤ .05) and restored to wild‐type levels when complemented with a functional copy of the TA system

As shown in Figure 2A, when stationary phase cultures were incubated at room temperature, the metabolic activity of wild‐type cultures decreased gradually over 120 h. In contrast, the Δ1718/1719 mutant strain was more sensitive to sustained exposure to acidic pH and lost metabolic activity to a greater extent than the wild‐type, especially between 72 and 120 h. Complementation of the deletion mutant with a functional copy of D11S_1718‐1719 TA system restored metabolic activity to wild‐type levels. As shown in Figure 2B, the Δ1194/1195 mutant phenotype was similar to the wild‐type strain through 72 h but exhibited a significant decrease in metabolic activity relative to the wild‐type at the 96 h and 120 h time points. Complementation of the mutant restored metabolic activity to the wild‐type level. Interestingly, the OD_600_ of the cultures did not significantly decrease over the incubation period for any of the strains (data not shown), suggesting that cell lysis may not be occurring as metabolic activity decreases.

### 3.4 Δ1718‐1719 and Δ1194‐1195 mutants exhibit reduced biofilm growth

**Figure 3:**
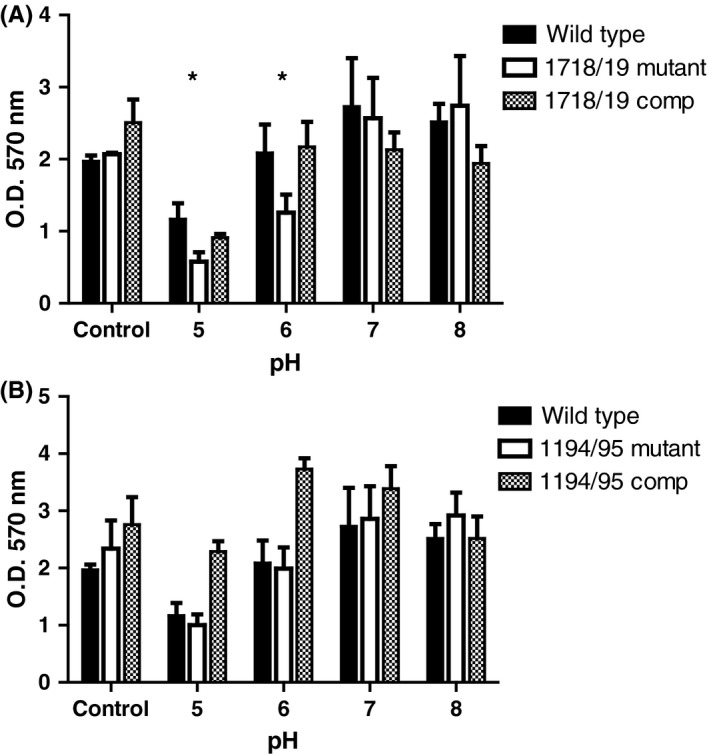
Biofilm biomass of wild‐type, toxin‐antitoxin (TA) system deletion mutants, and complemented strains under acidic conditions. Biofilms were initially grown for 72 h at pH 7.0 (Control), and subsequently incubated for an additional 24 h in fresh medium at pH 5.0 to pH 8.0. Biomass was quantified after staining with crystal violet. Asterisks indicate the conditions where biofilm biomass of the mutant strain was significantly reduced (*P *≤ .05) relative to the wild‐type and restored to near wild‐type levels after complementation

To determine if the D11S_1718‐1719 and D11S_1194‐1195 TA systems influence *A. actinomycetemcomitans* biofilm growth, static biofilms were grown for 72 h and then provided with fresh medium at pH 5.0 to pH 8.0. After incubation for an additional 24 h, biofilms were quantified by crystal violet staining. As shown in Figure 3A, biofilm biomass of the wild‐type strain increased after the addition of fresh medium at pH 6.0, 7.0 or 8.0, consistent with the previous findings of Bhattacharjee et al^41^ However, a significant decrease in biomass occurred when the wild‐type biofilm was incubated in fresh medium at pH 5.0. Biomass of the D11S_1718‐1719 mutant was significantly less than that of the wild‐type at pH 6.0 and pH 5.0, but was restored to wild‐type levels when the deletion strain was complemented with a functional copy of the TA system. Biomass of the D11S_1194‐1195 deletion mutant did not differ significantly from wild‐type; however, complementation of this strain resulted in a significant increase (*P *≤ .01) in biofilm biomass relative to wild‐type at pH 5.0 and 6.0, possibly arising from the presence of the TA system in multiple copy in the complemented strain. Together, these results suggest that the TA systems may contribute to the persistence of *A. actinomycetemcomitans* in mature biofilms exposed to acidic conditions.

### 3.5 D11S_1194‐1195 and D11S_1718‐1719 encode a ribonuclease and inhibit translation

**Figure 4:**
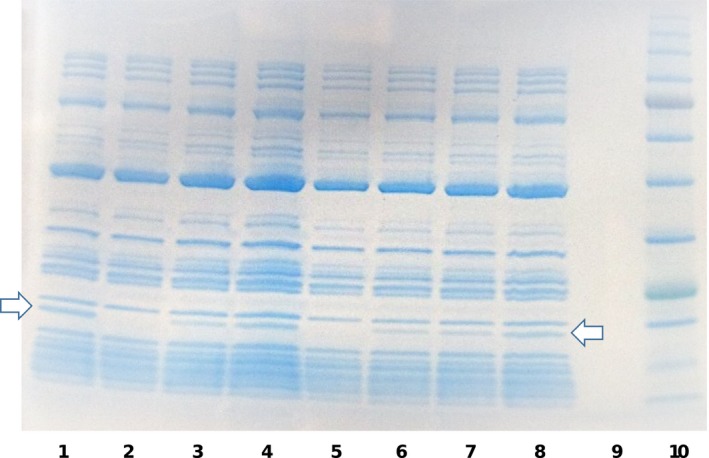
In vitro translation of dihydrofolate reductase (DHFR) in the presence of synthetic peptides representing the toxin and antitoxin proteins of the D11S_1718‐1719 and D11S_1194‐1195 toxin‐antitoxin (TA) systems. *In vitro* translation reactions were incubated with the toxin or antitoxin proteins of the TA systems, either alone or in combination and reaction products were electrophoresed in a Tris‐Glycine SDS‐PAGE gel. Lanes 1 and 8, positive control reaction without toxin or antitoxin protein (arrows indicates the DHFR protein); Lane 2, D11S_1194 toxin only; Lane 3, D11S_1195 antitoxin only; Lane 4, equal mixture of the D11S_1194‐1195 toxin and antitoxin; Lane 5, D11S_1718 toxin only; Lane 6, D11S_1719 antitoxin only; Lane 7, equal mixture of the D11S_1718‐1719 toxin and antitoxin; Lane 9, empty; Lane 10, size markers

To functionally characterize the D11S_1194‐1195 and D11S_1718‐1719 TA systems, each of the full‐length putative toxin proteins and their corresponding antitoxins was obtained as a synthetic peptide. These peptides were tested for inhibition of translation using the PURExpress In Vitro Protein Synthesis system using a control plasmid that expressed mRNA encoding DHFR. After conducting translation reactions in the presence and absence of the synthetic toxin and/or antitoxin proteins, samples were electrophoresed in a Tris‐Glycine SDS‐PAGE gel. As shown in Figure 4, DHFR (indicated by an arrow in Lanes 1 and 8) is readily produced in the control translation reaction in the absence of synthetic peptide. In contrast, no DHFR is produced when the translation reaction is conducted in the presence of the synthetic D11S_1194 toxin (Lane 2). DHFR production was unaffected by the synthetic D11S_1195 anti‐toxin peptide (Lane 3) or in the presence of equimolar amounts of both the synthetic D11S_1194‐1195 toxin and antitoxin (Lane 4). This result indicates that the synthetic D11S_1194 peptide functions as a toxin that inhibits DHFR translation and that the D11S_1195 protein represents its cognate antitoxin that is capable of preventing toxin activity when applied together. Similarly, synthetic D11S_1718 protein prevents translation of DHFR (Lane 5) whereas the D11S_1719 protein or an equimolar mixture of D11S_1718‐1719 proteins have no effect on DHFR production (Lanes 6 and 7, respectively). This result was unexpected as, from the structural organization of many RelBE TA operons, the open reading frame encoding the D11S_1719 protein would be predicted to encode the toxin component. In contrast, our results clearly show that D11S_1719 does not inhibit DHFR translation and so functions as an anti‐toxin and that the D11S_1718 peptide represents the toxin in this TA system.

**Figure 5:**
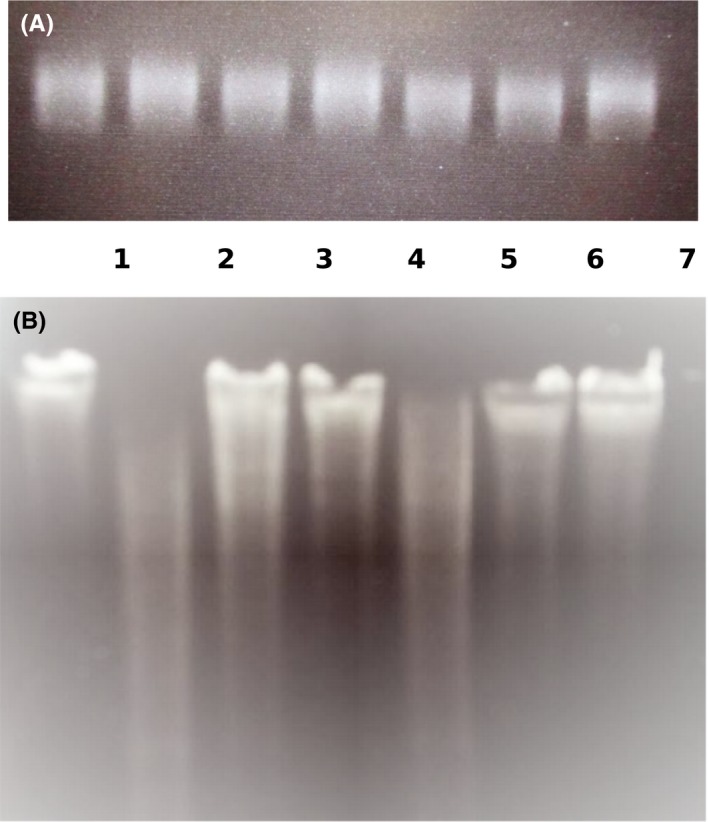
D11S_1194 and D11S_1718 toxins degrade MS2 RNA in the presence of ribosomes. RNA samples were incubated in the presence or absence of ribosomes and were analyzed on a TBE‐urea gel and stained with ethidium bromide. Lane 1, control, no toxin or antitoxin protein; Lane 2, D11S_1194 toxin only; Lane 3, D11S_1195 antitoxin only; Lane 4, equal mixture of the D11S_1194‐1195 toxin and antitoxin; Lane 5, D11S_1718 toxin only; Lane 6, D11S_1719 antitoxin only; Lane 7, equal mixture of the D11S_1718‐1719 toxin and antitoxin

To examine the mechanism of translation inhibition, we next determined if the toxin proteins function as a ribosome‐dependent ribonuclease. To accomplish this, MS2 virus RNA (MS2 contains a positive‐sense, single‐stranded RNA genome) was incubated with buffer (Figure 5A) or with purified ribosomes (Figure 5B) at 37°C for 25 min in the presence of the synthetic toxin and/or anti‐toxin proteins. As shown in Figure 5A, little degradation of MS2 RNA occurred in the absence of ribosomes. In contrast, as shown in Figure 5B, MS2 RNA was significantly degraded in the presence of ribosomes and synthetic D11S_1194 peptide (Lane 2) but not by D11S_1195 or an equimolar mixture of D11S_1194‐1195 peptides (Lanes 3 and 4, respectively). Similarly, addition of ribosomes and synthetic D11S_1718 toxin resulted in MS2 degradation (Lane 5) whereas little degradation was observed in the presence of D11S_1719 or a mixture of D11S_1718‐1719 proteins (Lanes 6 and 7, respectively). These results are consistent with the translation inhibition results and strongly suggest that the D11S_1194 and D11S_1718 toxins function as ribosome‐dependent endoribonucleases, which are inhibited by the D11S_1195 and D11S_1719 anti‐toxin proteins.

## 4 DISCUSSION

Type II TA systems are widely distributed in prokaryotes and increasing evidence indicates that they contribute to adaptation and persistence under conditions of stress.^21^, ^22^, ^23^, ^24^ The human oral cavity is an environmentally diverse niche that is populated by a complex microbial community comprised of over several hundred bacterial species,^10^, ^15^, ^16^ yet little is known about the role that type II TA systems play in the response of oral organisms to stress. *Aggregatibacter actinomycetemcomitans* D11S contains 14 TA loci that represent at least three of the six known families of type II TA systems. Consistent with the reported plasticity of the *A. actinomycetemcomitans* genome,^42^ three of these loci possess pseudogenes that have undergone significant gene deletion or rearrangement and are likely to be non‐functional. Only two of the TA systems identified in strain D11S_1 were not widely conserved across all seven *A. actinomycetemcomitans* serotypes. The HipAB‐like TA system was intact in only three of the 33 genomes examined (one serotype b and two serotype c stains), whereas 22 genomes possessed the antitoxin gene without the corresponding toxin gene. Most of the genome sequences analyzed were derived from strains isolated from humans, but many of the TA systems are also conserved in *A. actinomycetemcomitans* RhAA1, which was isolated from the oral cavity of a rhesus monkey.^43^ Indeed, the RhAA1 genome encodes a greater number of TA systems than the other *A. actinomycetemcomitans* genomes examined and many are present in multiple copies. Interestingly in *Mycobacterium tuberculosis*, the MazEF TA system is present in multicopy and the MazF toxins have been shown to be functionally distinct. Although MazF is known to function as a site‐specific mRNAse,^44^ two MazF toxins in *M. tuberculosis* also inhibit translation by cleaving at different sites in the 23S and 16S rRNAs^45^, ^46^ whereas a third MazF toxin cleaves tRNA^Pro^ and tRNA^Lys^.^47^ Hence, multicopy TA systems may provide several distinct mechanisms to respond to environmental stress and it is possible that the TA system content of the RhAA1 genome may reflect distinct mechanisms of adaptation to local environmental stress that exist in the monkey oral cavity.

Functional analysis of *A. actinomycetemcomitans* TA systems indicate that they respond selectively to a variety of environmental conditions to which the organism might be exposed in the human oral cavity or in the dental biofilm. *Aggregatibacter actinomycetemcomitans* readily colonizes and persists in the anaerobic niche of the subgingival pocket and indeed, seven of the type II TA systems were induced when *A. actinomycetemcomitans* was cultured under anaerobic conditions. This suggests that a high level of functional redundancy exists in the response and adaptation to anaerobic growth. Multiple TA systems of other human pathogens have also been shown to respond to specific host‐derived or host‐induced environmental conditions.^48^, ^49^ For example, 14 TA systems of *Salmonella enterica* serovar Typhimurium contribute to the intracellular formation of persister cells in macrophages^50^ and seven RelBE systems are induced in *Vibrio cholera* under virulence inducing conditions.^51^

In contrast, a more limited response was observed when *A. actinomycetemcomitans* was exposed to other environmental stresses such as reduced pH or iron limitation. D11S_1194‐1195 and D11S_1718‐1719 were highly induced when *A. actinomycetemcomitans* was exposed to acidic conditions similar to the conditions of a stationary‐phase broth culture. Deletion of D11S_1718‐1719 resulted in a significant reduction in metabolic activity of stationary‐phase cells relative to the wild‐type strain. In contrast, the phenotype exhibited by the Δ1194‐1195 stain was similar to wild‐type even though both the D11S_1718 and D11S_1194 toxin proteins function to inhibit translation and degrade mRNA (see below). One possible explanation for this result may be that activation of the D11S_1718 toxin functionally complements the loss of D11S_1194 toxin activity but D11S_1194 does not or only partially complements D11S_1718 function. Interestingly, although construction of the single TA deletion mutants was readily accomplished, multiple attempts to construct a 1194/1195‐1718/1719 double mutant failed, suggesting that the loss of both TA systems may be lethal to *A. actinomycetemcomitans*. Consistent with this, D11S_1718‐1719 was present and intact in all 33 *A. actinomycetemcomitans* genomes that were analyzed, whereas several genomes lacked D11S_1194‐1195, which might be tolerated if the D11S_1718 toxin functionally complements the loss of D11S_1194.

Many of the toxins encoded by RelBE family TA systems function as ribosome‐dependent ribonucleases that bind to the A site on the ribosome to cleave mRNA, so inhibiting translation.^52^ Our results indicate that the D11S_1194 and D11S_1718 toxins function similarly to other RelE toxins. Based on the gene organization of other RelBE family TA operons, in which the antitoxin gene precedes the toxin gene, our initial prediction was that D11S_1718 encoded the antitoxin protein and D11S_1719 the toxin. However, our results clearly showed that the D11S_1718 protein inhibits translation and has ribonuclease activity, indicating that it represents the toxin of this TA system. Hence, the D11S_1718‐1719 operon is organized differently from many other RelEB TA operons and is structurally similar to the MqsRA TA system that regulates biofilm formation by *E. coli*, where MqsR encodes the toxin protein.^53^ The physiologic outcomes that arise from activation of the D11S_1194‐1195 and D11S_1718‐1719 TA systems in vivo are not clear. TA system activation is associated with the formation of persister cells and is also important for biofilm formation.^20^, ^21^, ^22^, ^23^, ^24^ *Aggregatibacter actinomycetemcomitans* thrives in a complex multispecies biofilm in the oral cavity and in this context, *A. actinomycetemcomitans* has been shown to closely associate with commensal oral streptococci and benefits from a cross feeding relationship in which streptococci metabolize sugars to produce lactate, which serves as a primary energy source for *A. actinomycetemcomitans*.^54^, ^55^ However, *A. actinomycetemcomitans* is also acid sensitive and grows poorly in an acidic environment.^41^ It is possible that the activation of D11S_1194‐1195 and D11S_1718‐1719 at low pH facilitates this interspecies relationship by allowing *A. actinomycetemcomitans* to persist during periods of active streptococcal metabolism.

In summary, we have identified type II TA systems representing the RelBE, MazEF and HipAB families that are conserved across all seven serotypes of *A. actinomycetemcomitans*. Two systems, D11S_1194‐1195 and D11S_1718‐1719 respond to low pH and deletion of D11S_1718‐1719 results in reduced metabolic activity in *A. actinomycetemcomitans* and reduced biofilm biomass upon prolonged exposure to acidic conditions. Both TA systems function to inhibit translation by cleaving mRNA associated with the ribosomes and may represent novel therapeutic targets to control *A. actinomycetemcomitans* infections.

## CONFLICT OF INTEREST

The authors declare no conflicts of interest.
