# Supplementary material for: Identification and functional characterization of type II toxin/antitoxin systems in Aggregatibacter actinomycetemcomitans
Source: Mol Oral Microbiol. 2018 Feb 20;33(3):224–33. doi: 10.1111/omi.12215 (PMC5969271; doi:10.1111/omi.12215)
Supplement: Supplementary file 1 [file OMI-33-224-s001.docx]

| **Table S1. Bacterial strains used in this study** | | | |
| --- | --- | --- | --- |
| **Bacterial strain** | **Description** | | **Source** |
| 652 | wild-type,serotype C | Laboratory Strain | |
| 652-BWS1 | 652 Δ1194-1195 | *This study* | |
| 652-BWS2 | 652 Δ1718-1719 | *This study* | |
| 652-BWS1C | 652 Δ1194-1195::1194-1195 | *This study* | |
| 652-BWS2C | 652 Δ1718-1719::1718-1719 | *This study* | |
| XL1-Blue MRF^1^ | Δ(mcrA)183 Δ(mcrCB-hsdmSMR-mrr)173 endA1 supE44 thi-1 recA1 gyrA96 relA1 lac[F' proAB. lacIqZΔM15 Tn10 (Tcr)] | Stratagene | |
| One Shot Top10 | F- *mcrA* Δ( *mrr-hsd*RMS-*mcr*BC) Φ80*lac*ZΔM15 Δ *lac*X74 *rec*A1*ara*D139 Δ( *araleu*)7697 *gal*U *gal*K *rps*L (StrR) *end*A1 *nup*G | Thermo Fisher Scientific | |

|  |  |  |
| --- | --- | --- |

**Table S2. Plasmids used in this study**

| **Plasmid** | **Description** | **Source** |
| --- | --- | --- |
| pJT1 | Sp^R^ suicide vector | 33 |
| pJT4 | Km^R^ promoterless expression vector | 33 |
| pBWS1 | pJT1 derived, 1194-1195 | *This study* |
| pBWS1 | pJT1 derived, 1718-1719 | *This study* |
| pBWS1C | pJT4 derived, 1194-1195 | *This study* |
| pBWS2C | pJT4 derived, 1718-1719 | *This study* |
|  |  |  |
|  |  |  |
|  |  |  |
|  |  |  |
|  |  |  |
|  |  |  |
|  |  |  |

**Table S3. Composition of CDM medium**

| **Chemical/Compound** | **Concentration (mg/L)** | **Chemical/Compound** | **Concentration (mg/L)** |
| --- | --- | --- | --- |
| ZnSO_4_ | 0.7 | L-Serine | 100 |
| KI | 0.1 | L-Lysine HCl | 100 |
| CuSO_4_ | 0.065 | L-Histidine | 135.1 |
| Boric Acid | 0.5 | L-Glutamine | 100 |
| MgSO_4_ · 7 H_2_O | 700 | L-Asparagine | 113.6 |
| FeSO_4_ · 7 H_2_O | 5 | L-Methionine | 100 |
| MnSO_4_ | 5 | L-Isoleucine | 100 |
| NaCl | 100 | L-Proline | 100 |
| K_2_PO_4_ | 200 | L-Aspartic Acid | 100 |
| CaCl_2_ | 100 | L-Phenylalanine | 100 |
| KH_2_PO_4_ | 1000 | L-Tyrosine | 20 |
| NaMo_4_ | 0.5 | L-Cystine | 5 |
| KNO_3_ | 100 | L-Ornithine HCl | 20 |
| L-Glutamic Acid | 249.6 | L-Hydroxyproline | 20 |
| DL-Alanine | 200 | Adenine | 12.5 |
| L-Leucine | 100 | Guanine | 10 |
| Glycine | 100 | Cytosine | 13.5 |
| L-Valine | 100 | Thymine | 10 |
| L-Tryptophan | 100 | Xanthine | 10 |
| L-Threonine | 100 | Hypoxanthine | 10 |
|  | | | |
|  | | | |
| **Table S3. CDM Composition** | | | |
| **Chemical/Compound** | **Concentration (mg/L)** | **Chemical/Compound** | **Concentration (mg/L)** |
| Uracil | 10 | Vitamin B12 | 0.01 |
| Choline Chloride | 50 | NaHCO_3_ | 1000 |
| Β-Alanine | 10 | L-Cysteine | 650 |
| Pyridoxal | 1 | Glucose | 3600 |
| Pyridoxine HCl | 1 |  |  |
| Pyridoxamine · 2HCl | 1 | Magnesium L-Lactate* | 4050 |
| Spermidine · 3 HCl | 1 |  |  |
| Nicotinic Acid | 1 |  |  |
| Nicotinamide | 1 |  |  |
| Calcium Pentothenate | 1 |  |  |
| Spermine · 4 HCl | 1 |  |  |
| Thiamine HCl | 1 |  |  |
| Myo-inositol | 10 |  |  |
| Nicotinamide adenine dinuleotide | 1 |  |  |
| p-Aminobenzoic Acid | 0.1 |  |  |
| Pimelic Acid | 0.1 |  |  |
| Folic Acid | 1 |  |  |
| Riboflavin | 1 |  |  |
| D-Biotin | 0.1 |  |  |
| DL-6,8-Thioctic Acid amide | 0.1 | **MgSO_4_ was not added with Lactate* | |

| **Table S4. Protein sequences used for BLAST searches** | | |
| --- | --- | --- |
| **Toxin** | | **Toxin Sequence (protein)** |
| ccdB | mqfkvytykr esryrlfvdv qsdiidtpgr rmviplasar llsdkvsrel ypvvhigdes wrmmttqmvs vpvsvigeev adlshrendi knainlmfwg i | |
| parE | mlpvlwlesa dtdldditsy iarfdidaae rlwqrlrgcv lplsehpyly ppsdrvpglr eivahpnyii lyrvttssve vvnviharrq fp | |
| phD | mqsinfrtar gnlsevlnnv eageeveitr rgrepavivs katfeaykka aldaefaslf  dtldstnkel vnr | |
| mazF | mvsryvpdmg dliwvdfdpt kgseqaghrp avvlspfmyn nktgmclcvp cttqskgypf evvlsgqerd gvaladqvks iawrargatk kgtvapeelq likakinvli g | |
| chpK | mtrgeiwwvd lgipfgsepg fqrpvlivqn nafnhsnint iivvplttnl hlatapgnsm lkkedtnlsk dsivnvsqiv tidrerfikk vteiknkhmk kveegmklvl sles | |
| kid | xergeiwlvs ldptagheqq gtrpvlivtp aafnrvtrlp vvvpvtsggn fartagfavs ldgvgirttg vvrcdqprti dmkarggkrl ervpetimne vlgrlstilt | |
| pemK | mlkyqlknen gwmhrrlvrr ksdmergeiw lvsldptagh eqqgtrpvli vtpaafnrvt rlpvvvpvts ggnfartagf avsldgvgir ttgvvrcdqp rtidmkargg rlervpeti mnevlgrlst ilt | |
| chpBK | mvkkseferg divlvgfdpa sgheqqgagr palvlsvqaf nqlgmtlvap itqggnfary agfsvplhce egdvhgvvlv nqvrmmdlha rlakriglaa devveeallr lqavve | |
| relE | mayfldfder alkewrklgs tvreqlkkkl vevlesprie anklrgmpdc ykiklrssgy rlvyqvidek vvvfvisvgk rersevysea vkril | |
| yoeB | mkliwseesw ddylywqetd krivkkinel ikdtrrtpfe gkgkpeplkh nlsgfwsrri teehrlvyav tddslliaac ryhy | |
| ygjN | mhlitqkalk daaekypqhk telvalgnti akgyfkkpes lkavfpsldn fkyldkhyvf nvggnelrvv amvffesqkc yirevmthke ydfftavhrt kgkk | |
| yafO | mrvfktklir lqltaeelda ltadfisykr dgvlpdifgr dalyddsftw plikfervah ihlanennpf ppqlrqfsrt ndeahlvycq gafdeqawll iailkpephk lardnnqmhk igkmaeafrm rf | |
| ygiU | mekrtphtrl sqvkklvnag qvrttrsall nadelgldfd gmcnviigls esdfyksmtt ysdhtiwqdv yrprlvtgqv ylkitvihdv livsfkek | |
| mqsR | mekrtphtrl sqvkklvnag qvrttrsall nadelgldfd gmcnviigls esdfyksmtt ysdhtiwqdv yrprlvtgqv ylkitvihdv livsfkek | |
| yafQ | miqrdieysg qyskdvklaq krhkdmnklk ylmtllinnt lplpavykdh plqgswkgyr dahvepdwil iykltdkllr fertgthaal fg | |
| higB | mhlitqkalk daaekypqhk telvalgnti akgyfkkpes lkavfpsldn fkyldkhyvf nvggnelrvv amvffesqkc yirevmthke ydfftavhrt kgkk | |
| vapC | mldtnicsfi mreqpeallk hleqsvlrgh rivvsaitys emrfgatgpk asprhvqlvd afcerldavl pwdraavdat teikvalrla gtpigpndta iaghaiaaca ilvtnnvref ervpglvled wvr | |
| Zeta | manivnftdk qfenrlndnl eelvqgkkav esptafllgg qpgsgktslr saifeetqgn  vvvidndtfk qqhpnfdelv klyekdvvkh atpysnrmte alisrlsdqg ynlviegtgr ttdvpiqtat mlqakgyetk tyamavpkie sylgtierye tmyaddpmta ratpkqahdi vvknlptnle tlhktglfsd irlynregvk lyssletpsi spketlerel nrkvsgkeiq ptlerieqkm vqnqhqetpe fkaiqqkmes lqpptppipk tpklpgi | |
| hipA | mpklvtwmnn qrvgeltkla ngahtfkyap ewlasryarp lslslplqrg nitsdavfnf fdnllpdspi vrdrivkryh aksrqpfdll seigrdsvga vtlipedetv thpimawekl tearleevlt aykadiplgm ireendfris vagaqektal lrigndwcip kgitptthii klpigeirqp natldlsqsv dneyycllla kelglnvpda eiikagnvra laverfdrrw naertvllrl pqedmcqtfg lpssvkyesd ggpgiarima flmgssealk drydfmkfqv fqwligatdg haknfsvfiq aggsyrltpf ydiisafpvl ggtgihisdl klamglnask gkktaidkiy prhflatakv lrfpevqmhe ilsdfarmip aaldnvktsl ptdfpenvvt avesnvlrlh grlsreygsk | |
| hicA | mgktdkllak flnskktfew delvvlfssl gyvkkemqgs rvrffnaein htilmhrphp esyikggtlk aikqnlkeag ll | |
| yncN | mkqsefrrwl esqgvdvang snhlklrfhg rrsvmprhpc deikeplrka ilkqlgls | |

| **Table S5. *A. actinomycetemcomitans* genome sequences used in BLAST searches** | | |
| --- | --- | --- |
| **Strain** | **Serotype** | **GenBank Accession Number** |
| D7S-1 | A | CP003496.2 |
| H5P1 | A | AEJK00000000.2 |
| D17P-3 | A | ADOA00000000.2 |
| A160 | A | AJME00000000.2 |
| ANH9381 | B | CP003099.1 |
| HK1651 | B | CP007502.1 |
| Y4 | B | AMEN00000000.1 |
| RhAA1 | B | JPZI00000000.1 |
| SCC1398 | B | AEJP00000000.2 |
| I23C | B | AEJQ00000000.2 |
| SCC4092 | B | AJMF00000000.2 |
| S23A | B | AJMH00000000.2 |
| D11S-1 | C | CP001733.2 |
| SC383S | C | AZTR00000000.1 |
| SCC2302 | C | AEJR00000000.2 |
| D17P-2 | C | ADOB00000000.2 |
| AAS4A | C | AJMG00000000.2 |
| SA508 | D | AZTU00000000.1 |
| SA2200 | D | AZTY00000000.1 |
| SA269 | D | AZTX00000000.1 |
| SA3033 | D | AZTW00000000.1 |
| I63B | D | AEJL00000000.2 |
| SA3733 | D | AZTV00000000.1 |
| SC1083 | E | AEJM00000000.1 |
| SA2149 | E | AZTT00000000.1 |
| SC936 | E | AZTP00000000.1 |
| SA2876 | E | AZTS00000000.1 |
| ANH9776 | E | AZTZ00000000.1 |
| SA3096 | E | AZTQ00000000.1 |
| SCC393 | E | AEJN00000000.2 |
| SC29R | F | AZTO00000000.1 |
| D18P1 | F | AEJO00000000.2 |
| NUM 4039 | G | AP014520.1 |

| **Table S6. Primers used for pJT4 and pJT1 derived plasmids.** Sequences in bold indicate restriction enzyme sites for cloning. | | | |
| --- | --- | --- | --- |
|  | | | |
| **Primer** | **Primer Sequence (5’ – 3’)** | **Target** | **Product Size** |
|  |  |  |  |
|  |  |  |  |
|  |  |  |  |
| 11TA-rev | ATG TAA TGG ATA TGA ATT GGA TCC TTA TTT AAA CAC TTC CGA GTG |  |  |
| BWS1-115 | GTG GCA GCA GCT TAA CTA TGA TG**G** **CTA** **GC**T TAG TAC TCG CCG TCA TCA CCA TTT TAG CCA CCA TG | Upstream for 1194-1195 for pJT1 | 616 bp |
| BWS1-113 | CAC TGC GCT ATC TAA CAT AAT TTT TTC CTC CAT **GGG CCC** GGT GAA ATA |  |  |
| BWS2-115 | GGA AGT GTT TAA ATA A**GG** **GCC C**AA TTC ATA TCC ATT ACA TAT TAG | Downstream 1194-1195 for pJT1 | 575 bp |
| BWS2-113 | CAC GCC GTA GAA ACG ACG ATT TTC ATC AGA **CTG CAG** CAT GGC GGC AAT CGG |  |  |
| BWS3-175 | GAA AAG AGT GAG AAA ATG **GCT AGC** AAT CAA CAA CTT ATT GAT TTA AAA CAT AAG CTG GCG | Upstream 1718-1719 for pJT1 | 1120 bp |
| BWS3-173 | CGC AGA ATA ACT TAT TAC ATT **GGG CCC** CAT GTT ATT CTC C |  |  |
| BWS4-175 | CGC TAT CAC TAC TAA **GGG CCC** AAA ACG TTT AAT ATG TTC GAA CTC | Downstream 1718-1719 for pJT1 | 937 bp |
| BWS4-173 | GGA GCA TAG GTA AAG GCG CG**C** **TGC** **AG**C TAT AGA TAA ACA ATG CCT GTG |  |  |

**Table S7a. Conservation of TA systems in *A. actinomycetemcomitans* serotype a**

| **Strain** | **D11S_0150** | **D11S_0499** | **D11S_0906** | **D11S_0920** | **D11S_1023** | **D11S_1069** | **D11S_1144** | **D11S_1194** | **D11S_1418** | **D11S_1719** | **D11S_1799** | **D11S_2133** | **D11S_0469** | **D11S_2094** |
| --- | --- | --- | --- | --- | --- | --- | --- | --- | --- | --- | --- | --- | --- | --- |
| **D17P-3** | X | X | X | X | X X | O | X | X | X | X | X | X | X | X |
| **D7S-1** | X | X | X | X | X X | O | X | X | X | X | X | X | X | X |
| **H5P1** | X | X | X | X | X X | O | X | X | X | X | X | X | X | X |
| **A160** | X | X | X | X | X X | O | X | X | X | X | X | X | X | X |

O - only the anti-toxin gene is present

1 - putative pseudogene

**Table S7b. Conservation of TA systems in *A. actinomycetemcomitans* serotype b**

| **Strain** | **D11S_0150** | **D11S_0499** | **D11S_0906** | **D11S_0920** | **D11S_1023** | **D11S_1069** | **D11S_1144** | **D11S_1194** | **D11S_1418** | **D11S_1719** | **D11S_1799** | **D11S_2133** | **D11S_0469** | **D11S_2094** |
| --- | --- | --- | --- | --- | --- | --- | --- | --- | --- | --- | --- | --- | --- | --- |
| **ANH9381** | X | X X | X | X | X | X | X | X | X X | X | X X | X | X |  |
| **HK1651** | X | X X | X | X | X | X^1^ | X | X | X^1^ | X | X^1^ | X | X | X |
| **Y4** | X | X | X | X | X X | X^1^ | X | X | X X^1^ | X | X X^1^ | X | X | X |
| **RhAA1** | X | X | X | X X | X X X |  | X |  | X X | X | X X X | X X | X X |  |
| **SCC1398** | X | X X |  |  | X | X^1^ | X | X | X | X | X | X | X | X |
| **I23C** | X | X X | X | X | X | O | X | X | X | X | X | X | X | X |
| **SCC4092** | X | X X |  |  | X | O | X | X | X | X | X | X | X | X |
| **S23A** | X | X X | X | X | X | O | X | X | X | X | X | X | X | X |

O - only the anti-toxin gene is present

1 - putative pseudogene

**Table S7c. Conservation of TA systems in *A. actinomycetemcomitans* serotype c**

| **Strain** | **D11S_0150** | **D11S_0499** | **D11S_0906** | **D11S_0920** | **D11S_1023** | **D11S_1069** | **D11S_1144** | **D11S_1194** | **D11S_1418** | **D11S_1719** | **D11S_1799** | **D11S_2133** | **D11S_0469** | **D11S_2094** |
| --- | --- | --- | --- | --- | --- | --- | --- | --- | --- | --- | --- | --- | --- | --- |
| **D11S-1** | X^1^ | X^1^ | X | X | X | X | X | X | X | X | X | X^1^ | X | X |
| **SC383s** |  | X X | X | X | X | X | X | X | X X^1^ | X | X X^1^ | X | X | X |
| **SCC2302** |  | X X | X | X | X | O | X | X | X | X | X | X |  | X |
| **D17P-2** |  | X X | X | X | X | O | X | X | X | X | X X | X | X | X |
| **AAS4A** |  | X X | X | X | X | O | X | X | X | X | X | X |  | X |

O - only the anti-toxin gene is present

1 - putative pseudogene

**Table S7d. Conservation of TA systems in *A. actinomycetemcomitans* serotype d**

| **Strain** | **D11S_0150** | **D11S_0499** | **D11S_0906** | **D11S_0920** | **D11S_1023** | **D11S_1069** | **D11S_1144** | **D11S_1194** | **D11S_1418** | **D11S_1719** | **D11S_1799** | **D11S_2133** | **D11S_0469** | **D11S_2094** |
| --- | --- | --- | --- | --- | --- | --- | --- | --- | --- | --- | --- | --- | --- | --- |
| **SA508** | X | X | X | X | X X | O | X | X | X X^1^ | X | X X^1^ | X | X | X |
| **SA2200** | X | X | X |  | X X | O | X |  | X X^1^ | X | X X^1^ | X | X | X |
| **SA269** | X | X | X | X | X X | O | X | X | X X^1^ | X | X X^1^ | X | X | X |
| **SA3033** | X | X | X | X | X X | O | X | X | X X^1^ | X | X X^1^ | X | X | X |
| **I63B** | X | X | X X | X | X X |  | X | X | X | X | X | X | X | X |
| **SA3733** | X | X |  | X | X | O | X |  | X^1^ | X | X^1^ | X | X |  |

O - only the anti-toxin gene is present

1 - putative pseudogene

**Table S7e. Conservation of TA systems in *A. actinomycetemcomitans* serotype e, f and g**

| **Strain** | **D11S_0150** | **D11S_0499** | **D11S_0906** | **D11S_0920** | **D11S_1023** | **D11S_1069** | **D11S_1144** | **D11S_1194** | **D11S_1418** | **D11S_1719** | **D11S_1799** | **D11S_2133** | **D11S_0469** | **D11S_2094** |
| --- | --- | --- | --- | --- | --- | --- | --- | --- | --- | --- | --- | --- | --- | --- |
| **SC1083 (e)** | X^1^ | X X |  | X | X X X | X^1^ |  | X | X X | X | X X | X | X | X |
| **SA2149 (e)** |  | X X | X | X | X |  | X | X | X X^1^ | X | X^1^ | X | X | X |
| **SC936 (e)** | X | X X |  | X | X X X | O |  | X | X X | X | X X X^1^ | X | X | X |
| **SA2876 (e)** | X^1^ | X | X | X | X X | O | X | X | X X^1^ | X | X |  | X | X |
| **ANH9776 (e)** | X^1^ | X X |  |  | X |  |  | X | X | X | X | X^1^ | X | X |
| **SA3096 (e)** | X | X X |  | X | X X X | O |  | X | X X | X | X X | X | X | X |
| **SCC393 (e)** | X^1^ | X | X | X | X X | O | X | X | X | X | X | X | X | X |
| **SC29R (f)** | X | X | X |  | X X | O | X | X | X X^1^ | X | X X^1^ | X | X | X |
| **D18P1 (f)** | X | X | X | X | X X | O | X | X | X | X | X | X | X | X |
| **NUM4039 (g)** | X | X | X | X | X X | O | X | X | X X^1^ | X | X X^1^ | X | X | X |

O - only the anti-toxin gene is present

1 - putative pseudogene

**Table S8. Peptide sequences of D11S_1718-1719 and D11S_1194-1195**.

D11S_1718

MNVISYSAFRAELATTLDQVVADHSPVMITRQNGKHAVVMSLEDFAAYEETAYLLRSPKNRERLLASIDQLNSGKIIERELQE

m.w. - 9394.6

D11S_1719

MILAWTETAWEDYLYWQQVDKKTLLRINKLIQNITRAPFEGLGNPEPLKHQLSGFWSRRIDKEHRLVYQVSDSHLTIIQCRYHY

m.w. - 10,201.6

D11S_1194

MDYVLSKEYKRDLKKLPVEIQSGPEYAEVLYCLFNQKSLPERYKDHALQGNWQGFRDCHIKNDLILIYKIEADTLYFARLNSHSEVFK

m.w. - 10,515.9

D11S_1195

MLDSAVNFRTQADIKEQAFNVIKSYGLTPAQVLNMFLTQIAKTNTIPLSLDYQPNTKTANAINELMSGKGERFSVDSFDEFQQKMRDLSK

m.w. - 10,165.5
